# Clinical Spectrum and Trajectory of Innovative Therapeutic Interventions for Insomnia: A Perspective

**DOI:** 10.14336/AD.2022.1203

**Published:** 2023-08-01

**Authors:** Yun-Jo Lo, Viraj Krishna Mishra, Hung-Yao Lo, Navneet Kumar Dubey, Wen-Cheng Lo

**Affiliations:** ^1^EUDA Wellness, Taipei 114, Taiwan.; ^2^Biosource Tech, Ambala 133101, Haryana, India.; ^3^Victory Biotechnology Co., Ltd., Taipei 114757, Taiwan.; ^4^ShiNeo Technology Co., Ltd., New Taipei City 24262, Taiwan.; ^5^Department of Surgery, Division of Neurosurgery, School of Medicine, College of Medicine, Taipei Medical University, Taipei 11031, Taiwan.; ^6^ Department of Neurosurgery, Taipei Medical University Hospital, Taipei 11031, Taiwan.; ^7^Taipei Neuroscience Institute, Taipei Medical University, Taipei 11031, Taiwan.

**Keywords:** insomnia, cognitive behavioral therapy, whole-body cryotherapy, traditional chinese medicine, pulsed magnetic field therapy, acupressure

## Abstract

Increasing incidences of insomnia in adults, as well as the aging population, have been reported for their negative impact on the quality of life. Insomnia episodes may be associated with neurocognitive, musculoskeletal, cardiovascular, gastrointestinal, renal, hepatic, and metabolic disorders. Epidemiological evidence also revealed the association of insomnia with oncologic and asthmatic complications, which has been indicated as bidirectional. Two therapeutic approaches including cognitive behavioral therapy (CBT) and drugs-based therapies are being practiced for a long time. However, the adverse events associated with drugs limit their wide and long-term application. Further, Traditional Chinese medicine, acupressure, and pulsed magnetic field therapy may also provide therapeutic relief. Notably, the recently introduced cryotherapy has been demonstrated as a potential candidate for insomnia which could reduce pain, by suppressing oxidative stress and inflammation. It seems that the synergistic therapeutic approach of cryotherapy and the above-mentioned approaches might offer promising prospects to further improve efficacy and safety. Considering these facts, this perspective presents a comprehensive summary of recent advances in pathological aetiologies of insomnia including COVID-19, and its therapeutic management with a greater emphasis on cryotherapy.

Insomnia is one of the archaic but prevalent disorders causing sleep deprivation and impacting the quality of life, health, and economy throughout the world [1]. The prevalence of insomnia is more prominent in the elderly population and its progression depends on factors such as age, gender, behavior, environment, health conditions, the status of disorders, psychological and social conditions, etc. [2]. Additionally, the risk of insomnia may escalate up to three folds among cancer patients [3]. Incomplete sleep or recurring awakening during sleep are primary indicators of insomnia [4, 5]. The classical approach subdivides insomnia into three categories: i) Initial or pre-dormitional insomnia (delayed sleep), ii) Middle insomnia (fragmented sleep) and iii) Terminal or post-dormitional/ late insomnia-incomplete sleep [6]. However, various insomnia-related sleep characteristics have been grouped into the following four symptoms: (i) difficulty in initiating sleep (DIS), (ii) difficulty in maintaining sleep (DMS), (iii) early morning awakening (EMA), and non-restorative sleep (NRS) [7]. Acute insomnia mostly exerts short-term adverse effects, whereas chronic insomnia could potentially increase the risk of infection, depression, cardiovascular and respiratory disorders due to the impact of sleep disturbance on both innate and adaptive immunity [8-10]. Additionally, conditions such as comorbid insomnia and sleep apnea (COMISA), render difficulty in determining the exact etiology of insomnia [11]. Lack of sleep or disturbed sleep also deteriorates the condition of seizures due to neuroinflammation [12]. Insomnia disrupts the balance between sleep, the immune, and the central nervous system leading to inflammation and alteration in antiviral response [13]. Recently, the COVID-19 outbreak and related stress have been demonstrated to increase the risk of insomnia [14]. Therefore, sufficient sleep is highly crucial for hormonal balance and homeostatic immune functions for the defense of inflammatory activities, which is advantageous for long-term insomnia triggering systemic inflammatory disorders such as neurodegenerative disorders, diabetes, and atherosclerosis [15]. To address insomnia, current therapeutic interventions such as psychological, yoga, and meditation, traditional Chinese medicines and acupressure, as well as drugs have been explored [16]. However, these alternatives remain inadequate in overcoming insomnia [17]. Hence the novel electrostimulation-based approach such as cranial electrical stimulation (CES) is considered an effective candidate which mediates its therapeutic effect by regulating hormonal, neural, and behavioral responses [6]. Further, recent progress has shown that cryotherapy, in particular, whole-body cryotherapy (WBC) may exert a positive impact on disorders such as asthma, osteoarthritis, rheumatoid arthritis, pain, etc. [18]. WBC has also been evident in improving sleep quality, which opens the way to explore its therapeutic applicability for insomnia [19]. Based on the above-mentioned evidence, we have extensively reviewed pathophysiological aetiologies, incidence, and therapeutic progress in the treatment of insomnia.

**Figure 1.**
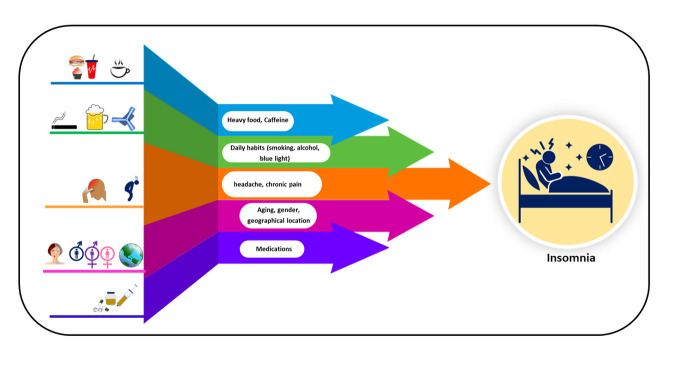
Etiolopathological factors of insomnia, which mainly involve heavy food, coffee, daily habits (smoking, alcohol, blue light), headache, chronic pain, aging, gender, and geographical location.

## Aetiology, epidemiology, and pathophysiology of insomnia

Insomnia is variably widespread throughout the world among all gender, age groups, and geographical locations which depends on factors such as stress, lifestyles, working culture, disease status, environment, sleeping conditions, etc. (Fig. 1)[20, 21]. The cases of insomnia have increased considerably and reached over 200 million in recent years [22]. The overall prevalence varies from 5% to 50%; however, the prevalence rate largely depends on the diagnostic criteria applied and the type or characteristics of insomnia [23]. The incidence of insomnia varies from 5.8 to 20% among the adult population in wider prospects [24], whereas, the prevalence of insomnia varies from 30% to 48% among the aging population [2]. This data could be under-reported due to lesser visits to seek medical assistance [25, 26]. Moreover, the recent change in lifestyle and excessive use of electronic devices have affected sleep quality and efficiency, thereby elevating the cases of sleep disturbances and insomnia [27-29]. The increasing dependency on mobile or computer screens for study, profession, social contact, and entertainment among youth, adolescents, and children is impacting their sleep quality [30-32]. Further, a cross-sectional study observed an association between insufficient physical activity and sleep problems [33]. The prevalence of insomnia is reported as 15.4%-33%, 15%, 24.6%-32.1%, 37%, 7%, 10.1%, 23.8%, 15%-29%, 3.0%-5.5%, 20.8%, 17%-23%, and 30-40% in the population of India [34-36], China [37], Sweden [38, 39], UK [40], Italy [41, 42], Europe [41], Canada, France [43, 44], Qatar [45], Spain, South Korea [46] and USA [47]. The factors which increase the prevalence of insomnia include response to social and economic factors, low income, and education level, susceptibility to chronic diseases such as osteoporosis, complications that arise due to menopause, etc. [48-51]. On a gender basis, the incidence of insomnia is higher among the female and aged population in Saudi Arabia, which may be associated with being elderly, widowed/divorced, females, or housewives, lack of education, and excessive tea consumption [52]. Moreover, ethnicity has a substantial and varying effect on insomnia prevalence and is considered a prominent factor in diagnosis [53, 54] This difference could be attributed to socioeconomic and lifestyle patterns of ethnic groups without any significant relation to genetic traits [55]. Furthermore, the COVID-19 pandemic has increased the prevalence of insomnia due to associated stress, anxiety, depression, fear, post-COVID-19 complications [56, 57].

**Figure 2.**
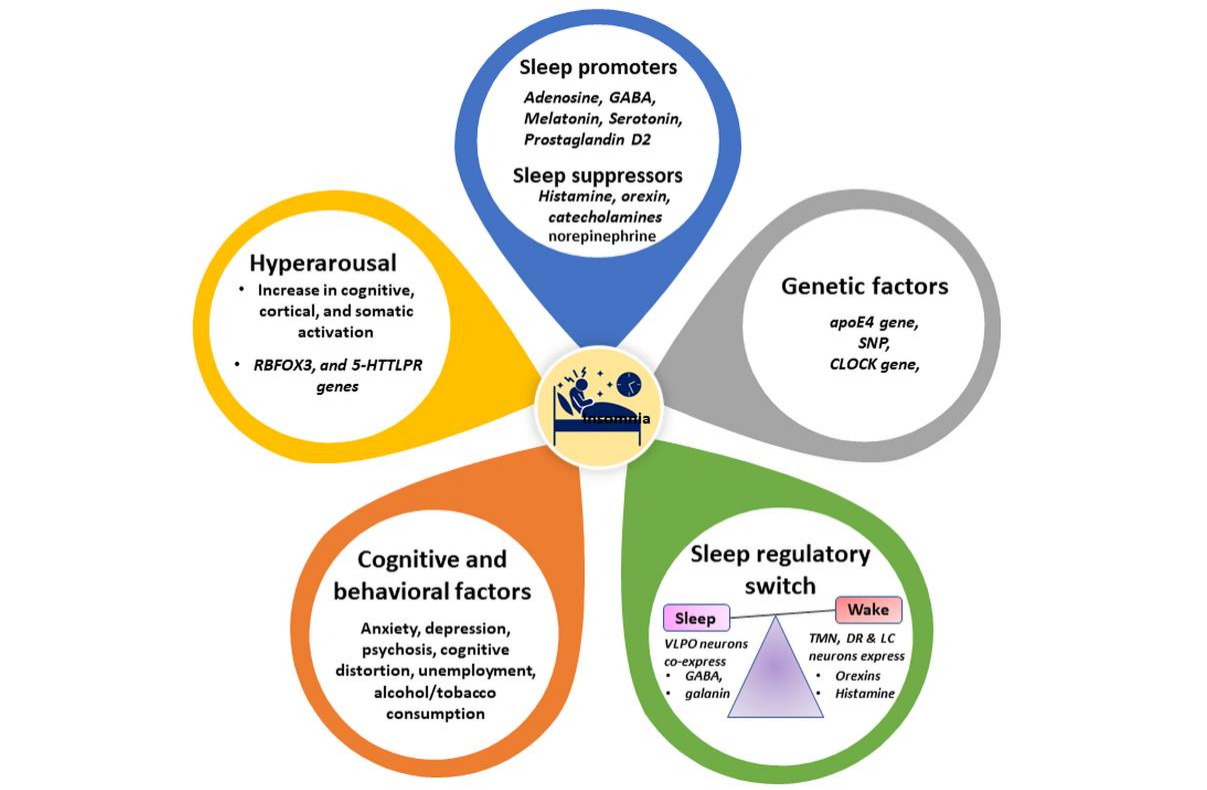
**Physiological factors contributing to insomnia**. Hyperarousal as a major contributor to insomnia may occur due to an increase in cognitive, cortical, and somatic activation, induction of RBFOX3, and 5-HTTLPR genes. Other factors include downregulation of sleep promoters (Adenosine, GABA, Melatonin, Serotonin, Prostaglandin D2) and upregulation of sleep suppressors (Histamine, orexin, catecholamines, and norepinephrine) which instigate sleep disturbance resulting in insomnia. The genetic factors (apoE gene, SNP, and CLOCK genes), cognitive, and behavioral factors (Anxiety, depression, psychosis, cognitive distortion, unemployment, and alcohol/tobacco consumption) also contribute to insomnia. Disturbance in sleep regulatory switch owing to molecular alterations associated with sleep (VLPO) and wake (*Orexins, TMN, DR, LC*) also results in insomnia symptoms. RBFOX3: RNA binding fox-1 homolog 3, 5-HTTLPR: Serotonin (5-HT) transporter polymorphism, GABA: Gamma-Aminobutyric Acid, apo E: apolipoprotein E, SNP: Single nucleotide polymorphisms, CLOCK: Circadian locomotor output cycles kaput protein, VLPO: Ventrolateral preoptic nucleus, TMN: Tuberomammillary nucleus, DR: dorsal raphe nucleus, LC: noradrenergic locus coeruleus.

Understanding the pathophysiology of insomnia is essential to developing effective therapeutic approaches. Insomnia is the result of an intricate communication of behavioral, physiological, and genetic factors [58] (Fig. 2). Multiple attempts have been made to develop models to interpret and explain the initiation and progression of insomnia [4]; however, are insufficient to express a comprehensive understanding. According to the neurocognitive model, the central character of insomnia is associated with hyperarousal, enhanced cognitive, cortical, and somatic activation [59-61]. The present models of hyperarousal and stress dysregulation explain the insomnia mechanism specifically by stimulating neurobiological and psychological activities triggering sleeping disturbance Intellectualtual or emotional cognitive arousal is associated with ruminative thinking leading to nocturnal awakening and hyperarousal [62]. Nocturnal arousal also causes depression and pre-sleep somatic arousal associated with insomnia. Additional activities such as computer gaming, internet surfing, and television watching impact sleep patterns resulting in a detrimental effect on the learning and memory of an adult [63-65]. Hence, even a half-hour of cognitive activity before sleep may induce emotional and mental flow resulting in sleep arousal and an increase in sleep period indicating early insomnia symptoms [23]. Further, the irregularity in sleep patterns such as prolongation of sleep latency, decrease in sleep efficiency, and sleep duration engender nocturnal cognitive arousal leading to insomnia and depression. However, cognitive arousal is more frequent during the night and could lead to physiologic hyperarousal [66]. The report evidenced that emotions either negative or positive, internal sleep locus control, and sleep belief are linked to varying impacts on sleep arousal in both genders [67]. It has also been noted that though hyperarousal is associated with positive and negative emotions in all gender, pre-sleep arousal is more inclined towards negative emotions among women.

In addition, physiologic hyperarousal has been reported in half of the chronic insomnia cases with an increased risk of diabetes, hypertension, and neurocognitive impairment leading to increased mortality [68, 69]. Moreover, severe physiological hyperarousal has been associated with underweight conditions. Furthermore, physiological hyperarousal-mediated insomnia triggers hypertension which has been confirmed by longer multiple sleep latency test (MSLT) values [70]. Physiologic hyperarousal also negatively impacts cognitive activity leading to an increase in error rate in different tasks [71]. Cortical hyperarousal is also an indicator of insomnia which is mainly triggered through pre-sleep cortical activity and causes delayed sleep onset [72]. Hyperarousal is indicated by the presence of symptoms such as increased heart rate, anxiety, anger or irritability, startle response, and struggle with relaxation [73, 74]. The higher brain activity and glucose metabolism in the wake center are recorded as evidence of hyperarousal among insomnia patients [75]. Failure of the arousal mechanism to trigger wake to sleep state could be linked to the incapacity to fall asleep. Low resilience triggers emotion, stress, and arousal dysregulation [76]. In summary, cognitive, physiological, and cortical hyperarousal has been reported as causative factors for insomnia, though extensive studies are required to decipher an exact mechanism underlying hyperarousal.

Recent research developments have paved the way to understanding the molecular mechanism and genes involved in the regulation of sleep, however, the exact pathways involved in insomnia have not yet been explored [77]. The predisposing, precipitating, and perpetuating factors representing the 3P model of insomnia indicate that inheritance and genetic factors are associated with insomnia and change in sleep pattern in response to stress. Sleep is primarily regulated through independent molecular mechanisms including sleep homeostasis and circadian rhythm. These two cellular pathways regulate the sleep/wake cycle under environmental conditions in association with light patterns [78]. Accumulation of adenosine and melatonin in the daytime primarily regulates sleep homeostasis and the circadian system [79].

Besides, the common methodologies including genome-wide association study (GWAS), family and twin studies along with candidate gene studies are being used to understand the role of genes in insomnia progression [80]. As per reports, the circadian gene such as *PER1, 2, 3, and CRY1* may be the genetic determinant of insomnia [81, 82]. The variable number of tandem-repeat (VNTR) polymorphism in *PER3* triggers sleep deficiency through the expression of the sleep homeostasis gene, therefore impacting sleep regulation and circadian insomnia-associated pathway [82, 83]. *PER 3* causes a change in light and cognitive responses due to altered brain activity and hypothalamic gene expression. The SNP in various genes has been studied to establish a correlation with insomnia; however, no strong association exists between them [84, 85]. Notwithstanding, SNPs such as rs10493596, rs2302729, rs12927162, rs324957/rs324981, and rs1823125 in genes *AK5*, *CACNA1C*, *TOX3*, neuropeptide S receptor (NPSR1), and PAX8, respectively contribute to insomnia symptoms [86]. In an integrative genome analysis, the abnormal expression level of genes including DALRD3, LDHA, HEBP2, TEX264, and FGFR3 have been identified as candidate genes contributing to insomnia, however, extensive studies are needed to establish their functional pathway [87]. The decreased levels of brain-derived neurotrophic factor (BDNF) during stress also trigger depression and sleep dysregulation leading to sleep deprivation or insomnia [88]. In response to stress, *RBFOX3,* a gene involved in the overexpression of neurotransmitter GABA, the ABCC9 gene in K_ATP_ channel, and the *5-HTTLPR* gene have been attributed to hyperarousal and wake promotion triggering insomnia [80]. Additionally, *apolipoprotein E4 (Apo E4)*, HLA-DQB1*0602, homozygous *Clock gene 3111C/C Clock,* and short (s-) allele of the *5-HTTLPR* has also been associated [89].

Biomolecules such as Orexins-A and Orexin-B regulate sleep-wake cycles. In presence of light, the orexin causes slow wave sleep and rapid eye movement and is responsible for the change of sleep to wake state [90, 91]. Orexins stimulate wake periods through activating orexin, cholinergic and monoaminergic neurons present in the hypothalamus/brainstem regions and specifically those expressed in the lateral hypothalamic area (LHA) [92, 93]. Notably, orexin neurons are distributed densely in the histaminergic tuberomammillary nucleus (TMN), serotonergic dorsal raphe nucleus (DR), and noradrenergic locus coeruleus (LC) which are involved in keeping individuals awake [92-95]. TMN and ventrolateral preoptic nucleus (VLPO) is the prominent region in the anterior and posterior hypothalamus which regulates the sleep-wakefulness cycle [96]. VLPO neurons are co-expressed with galanin and γ-aminobutyric acid (GABA) which triggers sleep, whereas TMN releases histamine involved in sustaining the wake stage [96, 97]. It has been demonstrated that synaptic suppression of the VLPO region disrupts the normal function of sleep-promoting neurons leading to increased wakefulness time [98]. VLPO seems to play a crucial role in sleep promotion, however, a recent study in VLPO lesion rat models demonstrated that VLPO might be involved in both sleep and arousal cycles [99]. Similarly, an increase in the level of catecholamines triggers disturbance and loss of nocturnal sleep [100]. The increase in the nocturnal level of norepinephrine is correlated with insomnia, indicating the significance of sleep in maintaining the function of the immune and sympathetic nervous systems [101]. Histamine also plays a crucial role in the control of the sleep/wake cycle. Specifically, the histamine receptors H1 and H3 play a crucial role in wake and sleep respectively, and the activation of the H3 receptor promotes sleep by downregulating the level of histamine [102].

γ-Aminobutyric acid (GABA) is a neurotransmitter inhibitor of the central nervous system (CNS) and regulates sleep by inhibiting neurotransmitter activity related to the wake cycle [103, 104]. Further, the increase in GABA level is considered an indicator of hyperarousal and its decreased levels in the anterior cingulate cortex (ACC) and medial prefrontal cortex (mPFC) have been associated with shorter sleep duration resulting in insomnia and impaired memory function [105]. Adenosine is a neuromodulator that accumulates during the wake cycle [106] and induces sleep by inhibiting GABAergic neurons in the ventrolateral preoptic area [107]. Further, chronic consumption of ethanol results in sleep disruption and remains sustained even after its termination by lowering ENT1 expression, adenosine, and glutamate transporter 1 resulting in the progression of insomnia [108]. Serotonin (5HT) plays a crucial role in sleep-wake regulation and its impact depends on receptors (5HT_1A_, 5HT_1B,_ 5HT_2,_ and 5HT_3_) and brain regions [109, 110]. Specifically, a decrease in 5HT may promote depression and insomnia among healthy individuals, which could be restored through therapeutic drugs [111]. Further, aerobic exercise exerts a positive impact on 5HT activities in the mid-brain and bloodstream and its release in the diencephalon and cerebrum promotes normal sleep [112]. Pineal gland-secreted melatonin during darkness maintains the circadian clock resulting in sleep stimulation and its regulation [113-115]. Reportedly, disruption in melatonin expression could also result in reduced immune response, sleep disturbance, and cancer [116]. Advancing age-dependent reduction of melatonin levels occurs mainly due to calcification of the pineal gland [117, 118]. Further, the prostaglandin D_2_ (PGD_2_) a lipid mediator which after being synthesized in the choroid plexus, leptomeninges, and oligodendrocytes region of the brain is secreted in cerebrospinal fluid, thereby inducing the adenosine accumulation in the hypothalamus leading to sleep induction [119]. The PGD2 in cerebrospinal fluid impacts D-type prostanoid receptors resulting in sleep induction, though the pathway by which PGD2 is cleared from the cerebrospinal fluid is unclear [120]. In addition to psychiatric disorders such as anxiety, depression, psychosis, and cognitive distortion, the factors which promote an abnormal increase in aminergic response, central aminergic state, and reduce the inhibitory neurotransmission could also be aetiologies of insomnia. [74, 121]. Thus, molecules such as orexins, GABA, 5-HT, melatonin, and PGD2 play a crucial role in sleep regulation and insomnia and have also been evaluated for their therapeutic candidacy for sleep disorders and insomnia. Besides these molecular factors, sociodemographic variables like higher education, unemployment, alcohol/tobacco consumption, and generalized anxiety disorders have also been significantly correlated with insomnia among the Indian population [122].

## Common Complications of Insomnia

**Figure 3.**
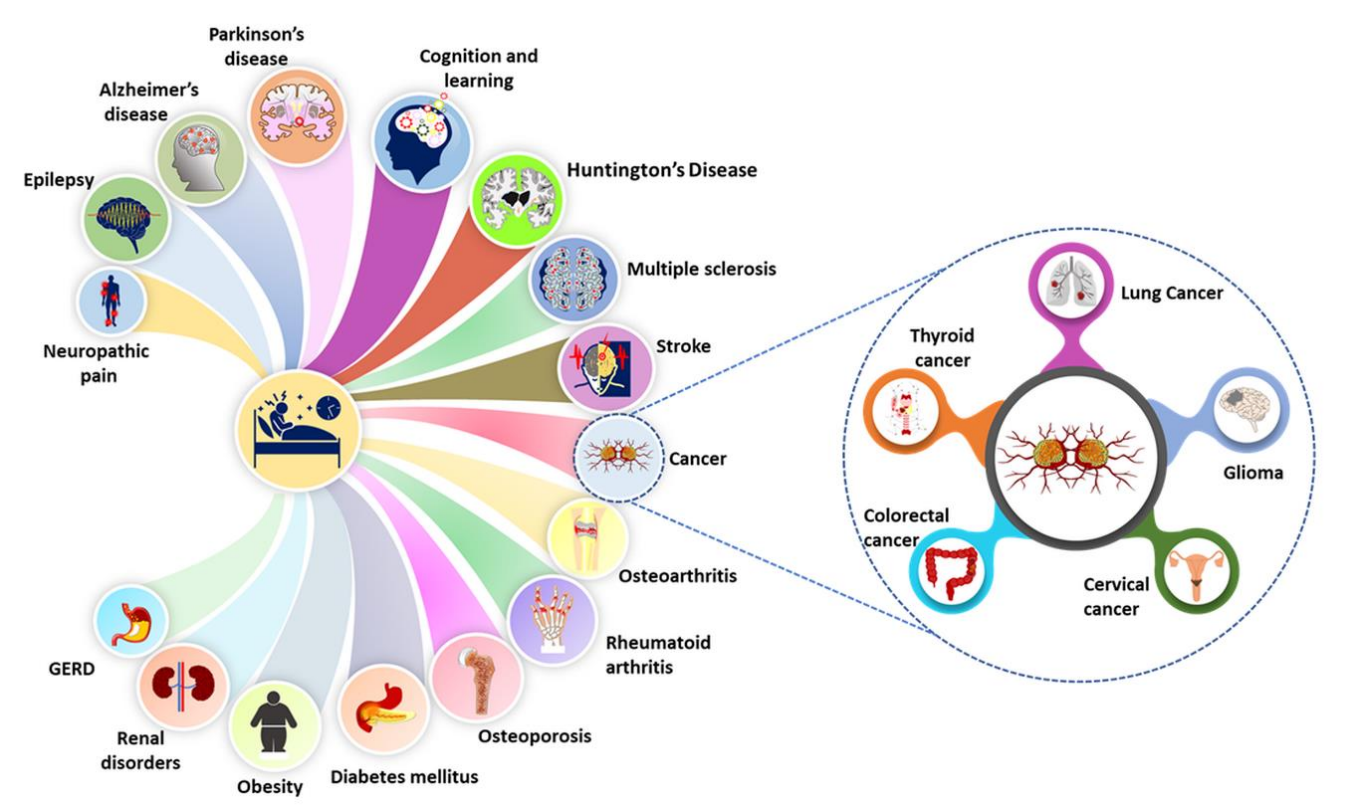
**Extent and health consequences of insomnia**. Possible mono- or bidirectional association between insomnia and other pathophysiologies including neurological (epilepsy, neuropathic pain, Alzheimer’s disease, Parkinson’s disease, cognitive and learning, Huntington’s disease, Multiple sclerosis), musculoskeletal (osteoarthritis, Rheumatoid arthritis, osteoporosis), metabolic (Diabetes mellitus, obesity, GERD), renal, and cancer (Glioma, thyroid, cervical, colorectal and lung). GERD: Gastroesophageal reflux disease.

Insomnia adversely impacts various organs (Fig. 3), resulting in aberrant sleep patterns, cognitive function, and emotional responses [123]. It also triggers neuropsychiatric disorders such as depression, dementia, mania, schizophrenia, and anxiety disorders [124], in addition to participating in the pathologic progression of the immune, endocrine and cardiovascular systems. It has been further reported that insomnia enhances the risk of hypertension, diabetes mellitus, arthritis, stomach ulcers, migraine, depression, obesity, heart attack/stroke, asthma, menstrual problem, obesity, and infection [123, 125, 126]. These disorders and complications have a cumulative impact on insomnia. Objective short sleep duration (OSSD) is a severe variant of insomnia and causes hyperarousal, hypertension, diabetes, variation in heart rate, neurocognitive disorder, induction of stress system, and increase in mortality [127]. It enhances the incidence of hypertension three times and is more prevalent than subjective short sleep [128, 129]. OSSD increases the risk of persistent insomnia though behavioral factors such as alcohol, caffeine, and cigarettes consumptions have not been significantly correlated [130]. Additionally, severe insomnia occurs with the deterioration of mental conditions, and OSSD of less than 6h is responsive to cognitive behavioral therapy [131]. Insomnia impacts telomere length; however, it varies according to gender, age, and ethnicity [132].

### Insomnia and neuropsychiatric disorders

Anxiety could be associated with fear/phobia, panic perception, separation, social discomfort, or attachments [121]. Multiple studies have indicated that anxiety with worry and extreme anxiety could be used as diagnostic indicators of insomnia [133-135]. A cross-sectional study confirms the relationship between anxiety and poor sleep quality among the Indonesian population [136]. Depression and insomnia are bidirectionally correlated, and both enhance the risk of each other [137, 138]. Major depression episodes are associated with symptoms of insomnia and hyper-insomnia, which increases the risk of recurrence of depression and suicidal attempts [139, 140]. It has been observed that regular insomnia could promote the late onset of depression and the promotion of major recurrent depressive disorders [141]. The directional association of insomnia, anxiety disorder, and depression has been studied and it was evident that prior anxiety is significantly associated with insomnia [142]. In a similar pattern, prior depression is not significantly associated with insomnia; however, prior insomnia could lead to severe depression. Nonetheless, more studies are required to establish the directional relationship between anxiety, insomnia, and depression.

### Insomnia and neurocognitive disorders

Prevalence of sleep disorders/insomnia has been observed among neurocognitive disorder patients. In particular, chronic insomnia, sleep apnoea and disrupted circadian rhythm has been correlated with Alzheimer’s, Parkinson’s disease, and Lewy body dementia [143]. The objective short sleep impedes cognitive functions and related cardiometabolic conditions [144, 145]. Sleep disturbance disrupts the balance between attention, memory, and the execution of cognitive controls [146]. Disturbed sleep might exacerbate neuropathic pain; however, a causal bidirectional association between them has also been implied [147, 148]. Neuropathic pain when associated with herpetic/post-traumatic trigeminal nerve dysfunction, or diabetes negatively impacts sleep quality resulting in characteristics of insomnia [147, 149-151]. Reports also indicate the association between sleep/awake state and epilepsy syndromes which has led to an acceptance of polysomnographic testing, a common diagnostic technique for monitoring epilepsy as well as comprehensive sleep [152]. Epilepsy patients also suffer from frequent insomnia with obviously increased morbidity and reduced quality of life (QoL) [153-156]. Poor sleep quality is strongly associated with the female gender due to overstress and gender discrimination [157, 158]. However, contrary studies also exist showing no significant difference in the frequency of insomnia on a gender basis [159]. Other factors such as social security and employment conditions negatively impact cognitive functions and the development of a sense of insecurity resulting in poor sleep [157]. Molecular studies have revealed that the dysregulated miRNAs in a patient with sleep-related epilepsy syndromes include miRNA-211, miRNA-155, miRNA-328, miR 194-5p, and miR 106b associated with CHRNA4, CHRNA2 CHRNB2 SCN1A, PAXNEB, GRIN2A, EFHC1, ClCN2, KCNQ3, KCNMB3, GABRA1, BRD2, KCNJ10, and CACNA1A [143, 151-159].

Besides, the stroke also increases the frequency of insomnia (one-third of stroke survivors) and is considered a prevalent complication that adversely impacts QoL, and mortality and enhances the risk of other secondary complications [160-163]. The association of post-stroke insomnia with depression, fatigue, and disability disrupts recovery and normal working life [163]. The incidence of insomnia is more prevalent among multiple sclerosis (MS) female patients than in normal populations and has been associated with MS-related fatigue [164, 165]. Sleep disturbance among MS patients could also be associated with depression, immunomodulatory therapy, body pain, and daytime sleep [166], and could contribute to memory loss [167]. Further, nearly 87.8% of Huntington's disease (HD) patients suffer from insomnia and other sleep disorders [168, 169]. The elevated motor function during sleep decreases rapid eye movement which results in short sleep duration and leg movement during sleep [168]. Nonetheless, sleep interference could be related to arousal associated with irregular movement rather than rapid eye movement [170]. Sleep disorders progressively worsen HD patients’ efficiency to perform daily routine/essential activities resulting in poor QoL [171]. The aberrant structural change and atrophy in brain regions such as the hypothalamus, locus coeruleus, and striatum could be associated with sleep disturbance among HD patients [171-175]. In the HD mice model, it has been demonstrated that rather than any direct impact from mutated huntingtin protein, the change in melatonin, cortisol, or disruption in circadian rhythms negatively impacts sleep [169, 176].

Sleep disturbance is also prevalent among glioma patients and the etiology of which could be multifactorial [177]. Insomnia could be associated with not only parenchyma damage, chemotherapy, and radiotherapy, but also with corticosteroids, antiepileptics, and psychoactive drugs administered to HD patients. Scientific evidence supports the role of glial cells in sleep homeostasis, determination of sleep period, regulation of damaged axons, and efflux of toxic content [178, 179]. These glial cell-mediated activities could be disrupted by insomnia [178]. A pre-clinical model suggests that chronic sleep loss accelerates the activation of microglia leading to astrocytes phagocytosis and risk of brain damage [180]. Chronic insomnia results in the increase of S100 calcium binding protein B and glial fibrillary acidic protein, whereas a decrease in BDNF and glial cell line-derived neurotrophic factor in serum is associated with pathological alteration in astrocytes leading to cognitive function loss [181]. These pieces of evidence indicate the adverse impact of insomnia on brain structure and function in terms of impaired cognitive function.

### Insomnia and Immune disorders

Sleep loss results in immune-associated disorders such as cardiovascular, autoimmune, and neurodegenerative diseases by negatively impacting both innate and adaptive immune systems by inducing inflammation [182]. It also reduces the immune response and therefore increases the risk of infection [183]. During chronic insomnia, the immune cell population of CD3^+^, CD4^+^, and CD8^+^ lymphocytes decreases, however without significantly affecting white blood cells, natural killer cells, and cytokine levels [184]. Further, sleep loss exacerbates the release of pro-inflammatory molecules C-reactive protein (CRP), tumor necrosis factor (TNF), IL-1β, IL-6, and IL-17, and promotes the progression of inflammatory disorders [182]. This occurs via induction of vascular endothelial molecules such as sICAM-1 and E-selectin along with activation of the nuclear factor kappa B (NF-κB) pathway [185-188]. Sleep deprivation induces the expression of signal transducer and activator of transcription (STAT) proteins (STAT1, STAT2, STAT3) in peripheral blood mononuclear cells (PBMC), monocyte, and lymphocyte populations resulting in activated innate immune arm and inflammation [189]. Sleep duration also plays a significant role in maintaining immune system balance; however, the associated pathways remain to be completely understood.

### Insomnia and carcinogenesis

In recent years, the association between insomnia/short sleep duration and cancer has also been investigated. A systematic review and meta-analysis by Shi et al. reported a 24% increase in cancer incidence with insomnia [190]. Specifically, the carcinogenic impact of nocturnal work and regular sleep disturbance has been evidenced in the terms of enhanced risk of advanced breast cancer, nasal neoplasm, lung cancer, colorectal adenoma, cervical carcinoma, and thyroid cancer [191-193]. This increased risk of malignancies might be associated with an immunocompromised state. While inducing carcinogenic events, insomnia initially inhibits melatonin synthesis, and disrupts immune function, circadian rhythm, and metabolism, leading to increased reactive oxygen species (ROS) [194]. Melatonin inhibits the uptake of tumor precursors-contained saturated and polyunsaturated fatty acids responsible for the synthesis of 13-hydroxyoctadecadienoic acid, a mitogen for linoleic acid-dependent tumor progression [194, 195]. Moreover, sleep deprivation also enhances DNA damage through the generation of ROS, thus elevating the risk of cancer. The disruption in circadian rhythm impacts angiogenesis, epithelial-mesenchymal transition, and KRAS signaling pathways and introduces the risk of bladder cancer and other malignant tumors [196]. Insomnia and fatigue could be indicators of cancer initiation and progression and need to be considered as a pre-diagnostic factor for screening cancer [193]. A Mendelian randomization study indicates the increased risk of lung adenocarcinoma and squamous cell lung cancer among insomnia patients [197]. A systematic review indicates that frequencies of insomnia are variably prevalent among different types of cancer patients in the range of 30%-93.1% [198, 199]. Insomnia also prevails in survivors of oral/head-neck cancer, and lung cancer [198, 200, 201], which might be attributed to stress, anxiety, depression, and pain, particularly among patients treated with chemotherapy [202]. It is of note that though scientific evidence is accumulating on the correlation between insomnia to cancer initiation and progression, the pathologic flow in the terms of mono-or bidirectional nature still needs to be investigated.

### Insomnia and musculoskeletal pathologies

It has been established that sleep loss also negatively affects bone mineral density (BMD) and its metabolism by altering the levels of sex/growth hormone and steroids, thereby inducing inflammation, another aberrant metabolism, and physical inactivity [203-206]. It has been evident that insomnia increases the risk of osteoporosis (7-fold) among aged women (age > 50 years) with a sleep duration of less than 5hrs/day [207]. Prominent bone loss may occur in postmenopausal women due to a lack of estrogen and the resultant increase in systemic inflammation [207-210]. A study among women indicates that short sleep, insomnia, or sleep disorders enlarge the scope of recurrent falls and body fractures except for the hip, which could be associated with a higher risk of osteoporosis [211, 212]. A population-based study asserts that short sleep duration significantly reduces the trabecular bone score (TBS) among obese adults and non-Hispanic women by adversely impacting the bone microarchitecture [213]. Thus, a combined low BMD and TBS representing the high frequency of osteoporotic fractures may be associated with insomnia [214]. The underlying possible mechanisms may include insomnia-mediated dysregulation of the hypothalamo-pituitary-adrenal (HPA) axis due to increased secretion of cortisol, altered levels of hormones (orexins, ghrelin, serotonin, melatonin), and stress-related metabolic alterations with its resultant impact on the sympathetic system [215].

Insomnia is also commonly found in osteoarthritis (OA) patients, possibly due to an increase in levels of systemic inflammatory cytokines [216]. Higher pain due to pro-inflammatory response in older OA patients has been shown to reduce with improvement in insomnia [217]. Another type of musculoskeletal disorder, known as rheumatoid arthritis (RA) has also been found significantly associated with insomnia, along with other factors such as depression, poor subjective health, and retirement [218]. A population-based survey indicates that pain and the urge to use the washroom may be the major cause of sleep fragmentation or disturbance among OA and RA patients [219]. Insomnia-mediated fatigue and emotional response such as anxiety and depression have an additive effect on pain under RA conditions [220]. The insomnia progression causes depression among RA patients due to an increase in inflammatory cytokines such as TNF-α, and cyclic citrullinated peptide, a rheumatoid factor [221].

### Insomnia and cardiovascular disorders (CVDs)

The current scientific studies have evidenced that insomnia exacerbates CVDs and increases the mortality and morbidity rate [222]. It enhances the risk of stroke and heart attack at a considerable level [223]. In a community-based cohort study, the risk of CVDs has been associated with poor sleep efficiency and a possible underlying mechanism could be linked to the activation of the HPA axis and nervous system resulting in increased blood pressure, urinary cortisol, and catecholamines levels, reduction/variation in heart rate which causes endothelial dysfunction and atherosclerosis [224, 225]. Besides, insomnia-mediated hypertension and diabetes could be associated with the induction of CVDs.

### Insomnia and asthma

Besides musculoskeletal deformities, insomnia severely worsens asthma characteristics along with sleep quality [226]. Thirty-seven percent of insomnia cases among asthmatic patients have shown increasing anxiety and depression and therefore a reduced quality of life [226]. The bronchial hyper-responsiveness increased bronchial resistance, and intense response to allergens escalate sleep disturbance among asthma patients [227]. In contrast to reports of bidirectional relationships, a positive unidirectional correlation between insomnia and asthma has been established using a genome-wide association study (GWAS) [228]. The study found that three shared genes i.e *ITPR3*, *HEXIM1,* and IQCH were involved between insomnia and asthma, while *ITPR3* and *HEXIM1* seem to be associated with an inflammatory response.

### Insomnia and gastrointestinal disorders

Alteration in cytokines (IL-1, IL-6, and TNF) levels also triggers gastrointestinal disorders such as gastroesophageal reflux disease (GERD), and inflammatory bowel disorder [229, 230]. A population-based study by Jansson et al. indicates that besides age, gender, obesity, tobacco/alcohol consumption, and social status, sleep-associated difficulty plays a vital role in the progression of GERD [231]. This may result from insomnia and sleep disorders' ability to lower salivary volume, the number of swallows, and frequencies of peristalsis [229]. The disruption in circadian rest-activity rhythms contributes to the progression of non-alcoholic fatty acid disorders, gastrointestinal, inflammatory, and irritable bowel disorders [232, 233].

### Insomnia and renal deficiencies

Insomnia also influences the sleep of kidney-transplanted patients by increasing stress, anxiety, and depression [234]. In the case of chronic insomnia, the risk of a high estimated glomerular filtration rate significantly increases with accelerated damage to kidney function; however, does not significantly contribute to end-stage renal stage disease (ESRD) [235]. However, the symptom of insomnia is also prominent among ESRD patients undergoing hemodialysis [236]. Hemodialysis is commonly associated with depression and its proper management can improve sleep quality. The increase in chronic kidney disease has also been associated with a sudden break in night sleep [237].

### Insomnia and liver disorders

Sleep disorders increase the risk of chronic renal failure and liver cirrhosis, and sleep abnormalities which is a general symptom of cirrhotic patients [238, 239]. Insomnia is a common symptom associated with liver cirrhosis [240], particularly in the presence of hepatic encephalopathy (HE) along with dysregulation of glucose and melatonin metabolism, thermoregulation, and ghrelin secretion [240]. Insomnia results in excessive daytime sleep (EDS) which enhances the risk of non-alcoholic fatty liver disease (NAFLD), a leading causative agent for chronic HE and liver cirrhosis [241]. The insomnia-mediated insulin resistance and metabolic syndrome could be correlated with the progression of NAFLD [241-243], possibly due to increased secretion of inflammatory cytokines such as IL-6 and TNF-α, leading to an abnormal increase in hepatic free fatty acids owing to adipocyte lipolysis [244-246]. Change in appetite behavior due to an increase in ghrelin and a decrease in leptin level promotes obesity, metabolic syndrome, lesser physical activity, and fatigue which significantly contribute to the progression of NAFLD [247-249]. Insomnia is also prevalent among hepatitis B virus-related liver disorder Chinese patients [250]. Based on the above-mentioned evidence, the condition of insomnia should be properly addressed to overcome its adverse impacts.

### Insomnia and metabolic syndrome

Sleep disorders disrupt the synergy of metabolism resulting in abnormal weight gain and obesity [251] through altering metabolic pathways triggered by the change in hormonal concentration such as leptin/ghrelin, appetite pattern, ANS response, HPA axis, and circadian rhythm which causes glucose tolerance and fat deposition. Sleep disturbance and circadian rhythm dysregulation may increase insulin resistance, glucose intolerance, and dysfunction of pancreatic β-cells leading to T2DM [252]. A cohort study indicates that insomnia, and short and long sleep duration is associated with obesity and central obesity [253]. However, though these recent studies have indicated the role of insomnia in abnormal weight gain, more in-depth studies are required to reach a consensus.

A population-based study documented that chronic insomnia plays a significant role in the progression of diabetes, while objective short-sleep duration might perpetuate the risk of cardiometabolic morbidity [254]. A bidirectional relationship between insomnia/short sleep and glucose metabolism, obesity, along with insulin resistance has also been indicated [255]. Sleep deficiency triggers deviation in metabolism pathways, food habits, and behavioral changes leading to metabolic complications [256]. Short sleep duration with slow wave sleep induces insulin resistance which diminishes glucose tolerance leading to type 2 diabetes mellitus (T2DM) development [257]. Reduced leptins and increased ghrelin levels, and the resultant inflammation occurring due to short sleep also play a crucial role in instigating T2DM [255-258]. The additional impact of sleep loss on food patterns includes an alteration in food sensitivity, and an increase in hunger and food consumption which promotes obesity and T2DM [259, 260]. Current studies indicate that insomnia has a significant role in the progression of T2DM; however, Green et al. pointed out that compared to short sleep duration, other factors such as smoking, lack of physical activity, alcohol consumption, obesity, and stress could also contribute to the diabetic condition [261]. Hence, the association between insomnia and diabetes needs to be extensively explored.

These aforementioned studies imply that pathological abnormalities associated with cancer, musculoskeletal, CVDs, asthma, gastrointestinal, renal, liver, and metabolic syndrome have been uni-or bi-directionally associated with insomnia and sleep disturbances. However, extensive studies are required to comprehend the molecular and biochemical pathways involved. While addressing therapeutic needs, it is essential to inhibit the progression of mild to chronic insomnia. Nowadays, cognitive behavioral therapy (CBT), pharmacological intervention with lifestyle changes, yoga, and exercise are commonly recommended.

## Insomnia: The Current therapeutic approaches

### CBT: A psychological intervention

CBT targets cognitive factors which contribute to the development of psychological abnormalities [262]. The primary objectives of CBT include a decrease in symptoms, functional recovery, and respite care. As an additive therapeutic approach, CBT is cognition, emotion, and behavior-specific [262, 263], and its ameliorative impact on depression, anxiety, stress, phobia, panic, and eating disorders has also been demonstrated [264, 265]. Based on the reported efficacy and safety of CBT in providing relief from various psychological disorders, it has also become an important non-pharmacological intervention for insomnia. CBT addresses insomnia-contributory factors such as regular diurnal napping, disturbed sleep-wake cycle, overconcern for poor sleep, anxiety, and overthinking [266]. Sleep restriction, stimulus control, and cognitive ordering through education are three basic elements of CBT [266, 267], which have shown a significant effect on sleep onset latency, sleep duration, sleep quality, sleep efficiency, and depression; however, its impact on pain has been found comparatively low [267]. CBT provides a relatively long-term effect lasting up to one year even after therapy [268]. A complex bidirectional relationship between insomnia and depression indicates that vestigial symptoms of depression after recovery could lead to the progression of insomnia [269-271]. The sequential treatment of insomnia and depression with CBT shows short-term efficacy, though long-term evaluation needed is needed to corroborate the therapeutic outcome [270]. CBT has also been found effective in controlling insomnia in OA pain and post-breast cancer survivors [272, 273]. In addition, compared to acupuncture therapy, CBT has been found as an effective candidate for primary care due to greater and long-term efficacy [274, 275].

Insomnia is associated with inflammation, as it impacts neurotransmitter levels, the HPA axis, oxidative stress, and mitochondrial activity [276-278]. The effect of CBT on suppressing inflammation has been demonstrated in depression [276] by lowering at least one of the inflammatory markers out of CRP, IL-6, and TNF-α [276, 279, 280]. A 12-week session of CBT effectively lowers the level of inflammatory mediators including IL-1Ra, IL-5, IL-6, IL-8, IL-10, granulocyte-colony stimulating factor, interferon-γ (IFN-γ), toll-like receptor 4 (TLR4), nuclear factor-κβ (NF- κβ) and TNF-α [281, 282]. Thus, CBT is promising in controlling inflammation and overcoming insomnia, however, treatment duration needs to be optimized. The therapeutic impact of CBT is mediated through its recruiting ability of emotion-regulatory circuits involving the ventromedial prefrontal cortex, anterior cingulate cortex, and amygdala leading to inhibited fear [283]. Thus, CBT has been established as a crucial therapeutic approach for insomnia and related disorders and should be extensively evaluated to recommend it as a primary treatment before prescribing medication.

### Pharmacological interventions

Therapeutic intervention mainly focuses on elevating sleep quality and duration along with improving daytime impairments [284]. If the initial psychological intervention does not respond sufficiently, the pharmacological approach is the immediate approach for the management. CBT has been associated with various limitations such as the unavailability of trained professionals, time-intensive and heavy cost, which lowers its wide application. Recent studies have also indicated its lesser efficacy on objective sleep parameters [285-287]. Thus, to provide immediate relief, easily accessible pharmaceuticals at lower cost with improved efficacy are preferred [284, 288, 289]. Short-term pharmacological treatment could also be supplemented with CBT [289]. Common drugs which are recommended for the treatment of insomnia are benzodiazepine receptor agonists (BZRAs) such as benzodiazepines (eszopiclone, zaleplon, and zolpidem), dual orexin receptor antagonists (suvorexant and lemborexant), sedative antidepressants (doxepin and trazodone), melatonin and melatonin receptor [285, 290-293]. BZRAs and Z-drugs are specifically preferred among older populations, however, their effect is weaker, short-termed, and is also associated with adverse effects such as the increased risk of falls and hip fractures cognitive impairment, daytime confusion, tolerance, and dependence [294, 295]. Further, though many insomnia patients are responsive to BZRAs, it has been evidenced that few patients are not remitted particularly in a comorbid condition that can further cause hypertension, and major depressive disorders [296]. To avoid BZRAs and Z-drug-associated adverse responses, new target sites such as orexin receptors are being explored [297]. Loss of orexinergic neurons has been associated with the condition of excessive sleepiness and narcolepsy [297]. Suvorexant has been reported as the first dual orexin receptor antagonist (DORA) which suppresses activation of the arousal system through reversible binding to orexin receptors. Not many neurophysiological disturbances or any other adverse effects of suvorexant exist except in a drowsy state [297, 298]. Though the associated adverse outcomes such as diurnal somnolence, unconscious nocturnal activity, suicidal ideation, and motor driving impairment have been reported at doses over 20 mg [297, 299]. Suvorexant treatment can improve total sleep time, sleep efficiency, insulin sensitivity, glycemic control, and inhibition of sympathetic activity among type 2 diabetes mellitus [300]. DORA is not being considered a replacement for BZRAs, however, these drugs have the potential to become a considerable alternative to BZRAs [301].

## Insomnia Treatment: Progress and novel approaches

Recent progress in insomnia treatments includes traditional and novel therapeutic approaches such as traditional Chinese herbs/medicine (TCH/TCM), acupuncture, pulsed magnetic field therapy (PMFT), and cryotherapy. In further sections, we have comprehensively summarized the developments of the above-mentioned approaches with a greater emphasis on cryotherapy in ameliorating insomnia.

### Traditional Chinese herbs (TCH)

TCH has been used for health promotion and prevention of diseases for a long time [302]. TCH alone or as an adjunctive therapy could be an effective tool to provide therapeutic relief among insomnia patients [303]. Wen dan tang, Suan Zao ren tang, Ban xia shu mi tang, suanzaoren, Fuling, Gancao, and Gui pi tang are the most recommended TCH formulation for insomnia [304, 305]. These herbs impart a sedative effect and improve insomnia through GABA or induction of the GABA receptor, suppression of the 5-hydroxytryptamine 1A receptor, and increase in orexin-A, orexin receptor-1, leptin, and leptin receptor level in the brain [305]. Suanzaoren decoction is one of the preferred choices for traditional treatment and its therapeutic potential could be associated with the regulation of Orexin-A, homeostasis of the HPA axis, and neurotransmitters [306]. Insomnia increases two-fold in depression which pushes mortality upward [307-309]. TCH and its formulations such as *banxia houpo*, *chaihu shugansan, Ge Gen*, *Huang Qin, Dan Shen*, *Bei Mu*, *Da Huang Ge-gen-tang*, *ganmaidazao, Kai-Xin-San*, *Ping-wei-san, sinisan*, *She Gan*, *shuganjieyu*, *Shu-jing-huo-xue-tang*, *Shao-yao-gan-cao-tang*, *wuling, xiaoyaosan*, and *yueju* are effective in the control of depression [309, 310]. Though these herbs might be effective, the herbal formulations are the preferred choice due to the additive effect and detoxication potential which increases the efficacy and safety of TCH [310]. The efficacy of TCH to address the therapeutic needs of insomnia and depression has been evident and could be an essential component of adjunctive therapy. A cohort study demonstrates that the use of TCH lowers the incidence rate of depression from 37.97 to 17.24 per 1000 among insomnia patients [307]. In the various randomized controlled trial (RCT), Suanzaoren decoction has been reported as effective and safe, though the incoherency in methods and fewer studies are limiting factors to establish any conclusion [311]. A meta-analysis found more efficacy of Chaihu Longgu Muli decoction (CLMD) than common drugs in reducing *Pittsburgh Sleep Quality Index* (PSQI) and improving total sleep time [312]. CLMD regulates the HPA axis, level of neurotransmitters such as norepinephrine, dopamine, and 5-hydroxytryptamine, suppression of adrenocorticotropic hormone and corticosterone, and inhibition of MEK/ERK pathways. Thus, TCH is considered an effective and safe alternative therapy for the treatment of insomnia and related disorders; however, its efficacy might be increased by supplementing other alternatives.

### Acupuncture

Acupuncture has been recognized as an effective nonpharmacological traditional intervention for neuroendocrinological disorders such as depression, menopause, and insomnia [313-315]. Short-term clinical impacts of acupuncture in lowering the Pittsburgh sleep quality index (PSQI), Zung self-rating anxiety scale, and Zung self-rating depression scale, indicates improvement in sleep quality, anxiety, and depression [316]. Acupuncture affects the neuroendocrine and immune systems by regulating neurotransmitters like serotonin, norepinephrine, dopamine, endorphins, and glucocorticoids [316, 317]. Traditional acupuncture improves sleep duration for a shorter duration [318], no significant effect impact has also been demonstrated in a case of residual insomnia associated with major depressive disorder [319]. Similarly, evidence shows that auricular acupuncture could be used for symptomatic treatment [320]. Of various acupuncture, scalp acupuncture and electroacupuncture (EA) have been more effective followed by warm and conventional acupuncture [321].

Approved by Food and drug administration (FDA), acupuncture therapy may overcome postoperative and chemotherapy adverse effects such as nausea, vomiting, myofascial pain, headache, low back pain, postoperative dental pain, asthma, fibromyalgia, and osteoarthritis [322-324]. Cancer-related insomnia, anxiety, and depression could be controlled through acupuncture; however, extensive studies are required to reach a consensus [322]. The underlying possible mechanism may be associated with a reduction in mitochondrial oxidative stress, HPA axis, and sympathetic nervous activity along with an increase in the level of ATP, gamma-aminobutyric acid (GABA), GABA(A) receptor, and melatonin [325-329]. Electroacupuncture (EA) inhibits glucose metabolism in the hypothalamus which decreases sympathetic stimulation [330, 331]. A pre-clinical study indicates that acupuncture lowers hippocampal neuronal loss, oxidative and inflammatory stress in vascular dementia by downregulating the expression of a thioredoxin-interacting protein involved in the activation of NOD-like receptor protein 3 (NLRP3) inflammasome [332]. Thus, acupuncture and EA have been reported for their potential impact on insomnia; however, the exact mechanism and concrete supportive evidence need to be further explored.

### Electro and pulsed magnetic field therapy (PMFT)

PMFT affects cellular water content, membrane structure and permeability, uptake of oxygen, and nutrients, mitochondrial function, and macrophage migration [333]. The application of alternating current (AC) affects the body’s electrolytes resulting in reversing of functional impairment via increasing blood circulation and cellular oxygen uptake. The magnetic field improves melatonin secretion from the pineal gland which restores circadian rhythm and sleep latency with the increase in sleep duration [333-335]. The impulse magnetic field has also been effective in providing relief from migraine and headaches [336]. In a double-blind controlled study, impulse magnetic field therapy has also demonstrated substantial or even complete relief from insomnia [333].

Further, transcranial magnetic stimulation (TMS), a non-invasive procedure that employs magnetic fields to stimulate nerve cells may depolarize neurons and modulate neuronal activity by generating an electric current [337, 338]. Repetitive TMS (rTMS) has also been found effective in neurological and psychological disorders. In a systematic review, its impact on sleep disorders has been documented; however, with inconsistent results [337].

Notably, BioBoosti, a device emitting a pulse of an electromagnetic field might invigorate endothelial and red blood cells by altering cell membrane charge and increasing microcirculation and perfusion [339]. Two weeks of treatment with BioBoosti significantly enhances slow-wave sleep and decreases night sleep disturbance resulting in improved sleep quality and insomnia symptoms. Interestingly, the magnetic stimulation at acupoints such as Neiguan (PC6), Shenmen (HT7), and Sanyinjiao (SP6) has also improved brain functional networking indicating improvement in sleep quality [340]. Thus, the treatment with a magnetic field seems to be a promising therapy for insomnia. However, extensive studies are needed to understand the comprehensive potential of magnetic therapy.

Electrotherapy is another perspective non-pharmacological therapeutic intervention for insomnia. In cranial electrotherapy stimulation (CES), the microcurrents of 1-1.5 mA affect specific nerve cells, and increase levels of neurotransmitters such as acetylcholine, adrenocorticotrophic hormone, serotonin, dopamine, β-endorphin, cholinesterase, and norepinephrine, and decreases cortisol, leading to increased flow of blood and cerebrospinal fluid [341-344]. These neurochemical alterations reduce pain, stress response, and spasticity, maintain homeostasis, and enhance mood, relaxation, immune function, pain tolerance, and pleasure resulting in improved sleep quality [344]. CES enhances alpha while reducing beta and delta activity associated with relaxation, ruminative thoughts, and fatigue, respectively. In addition, CSE also improves focus, concentration, and mental awareness by influencing beta regions [345]. CES decreases sleep latency and enhances sleep improvement with sleep quality through its implication in the treatment of depression and anxiety needs to be carefully evaluated [346]. CES shows satisfactory improvement in ESAS, HADS, BPI, and PSQI scores among cancer patients indicating relief from anxiety, depression, pain, and insomnia [347]. Sleep hygiene improves the efficacy of CES treatment and both approaches could be recommended together as adjunctive therapy [348]. Consequently, the CES, transcutaneous trigeminal electrical neuromodulation (TTEN) inhibits noradrenergic activity and could be enveloped as a novel treatment option [349]. CES is approved by US FDA and has been effective to control anxiety, depression, and insomnia, however, this treatment is considered the last option and has been associated with mild and limited adverse events such as vertigo, skin irritation, and headache.

### Cryotherapy

Cryotherapy was first used successfully to treat rheumatism in the 1970s [350, 351]. In recent years, cryotherapy has been extended to mental disorders, depression, anxiety, inflammatory disorders, pain, and muscle fatigue [352-357]. Cryotherapy has been classified into partial-body (PBC) and whole-body cryotherapy (WBC), and both methods expose the body partial or whole) at a very low temperature of -10°C to -170°C for durations between 20 s to 3 min in cryo-compartments such as cryo-chamber or cryo-cabin [358]. PBC focuses on a single individual and nitrogen is applied directly on body parts excluding the head and neck, whereas WBC involves exposure to extremely cold dry air (usually between -100°C and -140°C) for short durations generally between 2 to 5 minutes [358-360]. Taking this into consideration, even a few global companies including Euda wellness, a subsidiary company of Victory Biotechnology Co., Ltd. Taipei, Taiwan, are offering WBC for insomnia. The therapeutic potential of cryotherapy in pain has been associated with its potential to suppress oxidative stress, inflammation, edema, and neurotransmission in pain fibers [359, 361, 362], by lowering the level of cytokines such as IL-1, IL-6, IL-10, and TNF-α [363]. The WBC further improves the efficacy of kinesiotherapy two-folds resulting in improved pain and spinal movement among ankylosing spondylitis (AS) patients [364] by lowering CRP, fibrinogen, TNF- α, ICAM-1 as well as a considerable reduction in lipid profile, atherosclerosis plaque instability, oxidative stress, [365, 366]. The WBC reduces paraoxonase-1 activity, total oxidative status, inflammatory endothelium parameters, serum CD40L, and serum amyloid A resulting in decreased oxidative stress and a positive effect on human endothelial homeostasis [367]. Computerized cognitive training when applied synergically with WBC could improve cognitive and depressive functions [368, 369]. After cryotherapy, the blood flow in the skin and extremities improves and becomes richer in oxygen, and nutrients [370]. Cryotherapy also stimulates the production of endorphins and anti-inflammatory biomacromolecules [371]. First exposure to cryotherapy may be unpleasant, however, repeated exposure improves the treatment experience [370]. The effectiveness of cryotherapy in regulating cytokines, oxidative, stress inflammatory response, blood flow, oxygen level, fatigue, pain, depression, and anxiety provides an opportunity to improve sleep quality. In a small scale clinical trial, cryo-stimulation (partial body cryo-stimulation at -180 ^0^C for 180 seconds) among professional soccer players indicated a controlled movement in sleep and better sleep quality [372]. On the other hand, two weeks of regular cryo-stimulation exposure at -90^0^C improve somnolence and psychological behavior among the aged population with sleep disturbance and restless leg syndrome [373]. After exhausting training and tournament, the pro-inflammatory cytokines such as TNF-α, and IL-1β, increases which causes systemic inflammation leading to the development of the overreaching syndrome and poor performance [374-376]. A five-day WBC (-120 ^0^C) treatment twice a day can also significantly lowers TNF-α and increase IL-6, indicating the efficacy of cryotherapy in improving the performance of tennis players [376]. The 3-minute WBC improves sleep quality after exercise/training and improves pain relief by ameliorating parasympathetic nervous activity during the slow-wave sleep period [377]. Cryotherapy provides relief from depression and anxiety due to its positive impact on the HPA axis and endogenous opioids [356]. WBC enhances the therapeutic impact of anti-depressant and their response time [378]. In a randomized clinical trial, WBC significantly improved depressive symptoms, quality of life, and self-assessed mood which indicates its additive impact on pharmaceutical treatment [379]. Cryotherapy has also been found effective in tackling tension-type headaches (TTH) associated with stress and mental pressure among students [380]. The needle pain causes anxiety among children and impacts satisfaction during blood collection, which could be suppressed by cryotherapy resulting in increased satisfaction [381]. In addition, the combination of cryotherapy with vibration lowers the fear, anxiety, and anguish among children [382].

The potential of cryotherapy to reduce inflammation and pain has also been explored to assess its impact on OA and other types of arthritis. Cryotherapy with physical exercise further improves pain relief among OA patients [383]. A systematic review evidenced that cryotherapy improves knee pain and motion in OA; however without any significant impact on blood loss during knee replacement surgery [384]. In the rat model, cryotherapy has been demonstrated to decrease synovial fluid leukocyte count and inflammatory cytokine level resulting in improved footprint patterns [385]. Notably, no significant impact of short-term cryotherapy on pain, function, and quality of life improvement among knee OA patients has also been reported [386]. Cryotherapy improves pain in the anterior knee infusion model, though with no significant impact on vastus medialis motoneuron pool excitability [387]. A 10 cycle of WBC significantly lowers the frequency and pain level resulting in a decreased use of analgesics and improved physical activity and well-being [388]. Cryotherapy has been observed to lower synovial power-Doppler ultrasonographic (PDUS) activity and pain among OA patients [389]. The encouraging outcome of WBC has also been observed among RA patients by reducing the level of pro-inflammatory molecules such as IL-6 and TNF-α [390], possibly by decreasing the level of total peroxyl radical trapping antioxidant capacity of plasma [391]. WBC ceases inflammation, and pain along with functional recovery among RA patients which could be associated with its detrimental impact on histamine secretion and degradation [392]. The therapeutic efficacy of cryotherapy could also be correlated with its impact on improving microcirculation, muscle tension, hormonal, and immune response along with analgesic, anti-oedematous and anti-inflammatory effects [388, 393-396]. Oxidative stress is a characteristic phenomenon in neurodegenerative disorders and could be associated with inflammatory reactions [360, 397, 398]. WBC possesses the potential to modulate systemic oxidative stress among MS patients by increasing total antioxidative status [398, 399]. Fatigue in MS could also be a causative agent for insomnia which can be overcome with combined therapy of WBC and kinesiotherapy [400].

The cognitive dysfunction due to insomnia could be addressed through cryotherapy which has become a potent tool for early stage of mild cognitive impairment (MCI) [401]. The progression of MCI to dementia is well-established and up to 64% of patients suffer from dementia [402]. The efficacy of the pharmacological intervention in improving mental health/disorders such as depression and QoL could be further improved by add-on cryotherapy [403, 404]. Decreased level of nitric oxide indicates endothelial dysfunction and oxidative stress, WBC improves inducible nitric oxide synthase though no significant effect has been observed on nitric-oxidative stress or inflammation markers [405]. Cryotherapy possesses the potential to alter hormonal and lipid profiles contributing to its anti-inflammatory and oxidative role which hinders the etiopathogenesis of dementia [390]. Systemic cryotherapy lowers rheumatic activity and related pain; however, no significant change has been observed in the levels of pro-inflammatory and anti-inflammatory cytokines [406]. It has also been observed that cryotherapy significantly improves locomotor activity resulting in encouragement to work and positive self-assessment [407].

Cryosurgery is a minimally invasive approach that is frequently used in the treatment of metastatic liver and prostate cancer [408]. Though the therapy is normally followed by chemo- or radiotherapy to improve its efficacy, it is associated with freeze injury, coagulative necrosis, cell membrane damage, local blood vessel thrombosis, and apoptosis [408, 409]. Cryotherapy is promising in the treatment of liver cancer without any significant bile leakage or mortality, [410]. in combination with low-level laser therapy has been found effective in cancer patients with oral mucositis [411] or bladder cancer [412]. Further, percutaneous cryoablation provides progression-free survival without any major adverse events and a significant reduction in hematuria, urinary irritation, hypogastralgia, and lumbago [413]. The cryotherapy intervention improves insomnia and associated disorders resulting in ameliorated QoL; These scientific reports are evident in exploring the therapeutic role of cryotherapy; however, extensive studies are required to establish the underlying mechanistic insight.

## Biomaterials, stem cells and insomnia

Traditional drug delivery restricts the therapeutic outcomes which necessitate the development of novel, effective and safer alternatives [414]. Biomaterials such as biopolymers and synthetic biopolymers have been widely explored in the controlled transdermal, intranasal, and transbuccal delivery of drugs [414, 415]. The oral uptake of melatonin and other insomnia drugs results in low bioavailability, and limited efficacy [416, 417]. To overcome the low bioavailability, various attempts have been made to develop drug delivery systems. A single-walled carbon nanotube (SWCN) improved bio-absorption of zaleplon (sedative-hypnotic) via nasal delivery, by increasing carbon nanotube-mediated blood-brain crossing ability [417], leading to prolonged neuroprotective effect. Melatonin is another suitable therapeutic candidate for insomnia which could be transdermally delivered due to its low molecular weight [415, 418-420]. A microneedle patch synthesized from proline, melatonin and silk fibroin has been found effective in maintaining drug concentration in blood and demonstrates physiological resemblance in drug release in *Sprague-Dawley rat* models of insomnia [416]. Moreover, soya phosphatidylcholine and sodium deoxycholate-based elastic liposomes may facilitate melatonin release by enhancing transdermal flux and reducing lag time; therefore, could be helpful in improving insomnia [415]. Thus, biomaterials-based drug delivery seems an effective approach to better the therapeutic outcomes of insomnia drugs. However, more extensive clinical and scientific studies are required to select the most effective and safe delivery systems.

Another regenerative biomaterial therapies include stem cells, which have become a prominent choice for treatment of various degenerative disorders. Sleep fragmentation has also been correlated with stem cells. Specifically, it impacts the immunity and functioning of hematopoietic stem cells (HSPC) through epigenetic modification, leading to aging of hematopoietic system and collapse of clonal diversity and homogenization of myeloid pool [421]. Notably, insomnia-related genes such as MADD, CASP9, PLEKHM2 have been found significantly enriched in neural stem cells (NSCs) [422]. The NSC also plays a key role in genesis of sleep-waking cycle switch-associated neurons. Though no direct studies exist, this above-mentioned evidence indicates that stem cells therapies may be helpful in generation of sleep-related neurons. Consequently, this may also assist in the improvement of insomnia associated complications.

**Figure 4.**
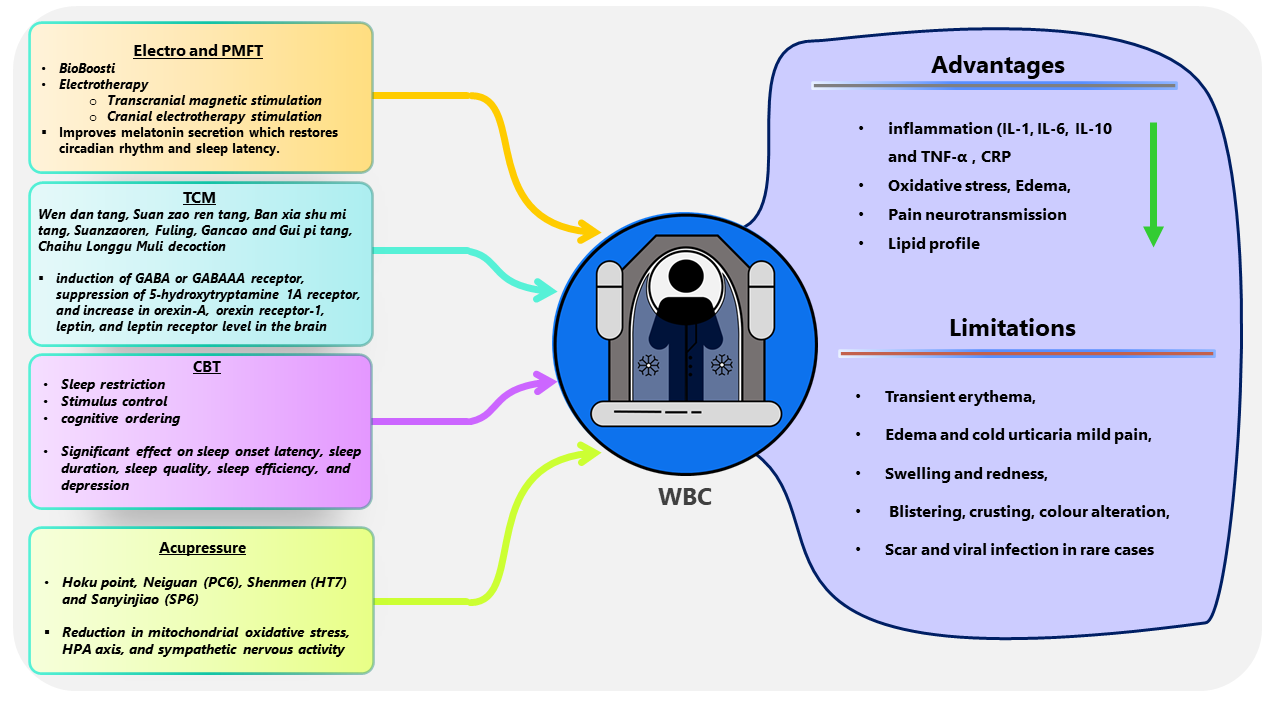
**Possible synergistic application of whole-body therapy and other alternatives including PMFT, TCM, CBT, and acupressure for insomnia**. This combination therapy may decrease the level of inflammatory molecules (IL-1, IL-6, IL-10, TNF-α, and CRP), oxidative stress, edema, pain neurotransmission, and lipid profile. The limitations of cryotherapy include transient erythema, edema, cold urticaria, mild pain, swelling and redness, blistering, crusting, color alteration, scar, and viral infection in rare cases, which might be recovered through other combinational therapeutic alternatives. TCM: Traditional Chinese Medicine, CBT: Cognitive behavioral therapy, PMFT: Electro- and Pulsed Magnetic Field therapy. IL-Interleukin, TNF: Tumour necrosis factor, CRP: C-reactive protein.

## Prospects of Combinatorial cryotherapy: Future directions for augmenting therapeutic impacts

Cryotherapy in combination with other therapeutic approaches implies the potential to improve insomnia symptoms (Fig. 4). It has been applied as an add-on therapy to pharmacological treatment for the treatment of depression [379] by regulating pain, inflammation, oxidative stress, immune response, and the circulation system [356]. WBC's role in enhancing the level of pro-inflammatory cytokines and lowering the circulation of pro-inflammatory molecules along with its potential to regulate the *hypothalamic-pituitary-adrenal (*HPA) axis could support pharmacological outcomes of drugs in insomnia treatment. Moreover, the adverse effect of pharmaceutics such as cognitive impairment, and daytime confusion could be addressed by cryotherapy [401, 423]. Insomnia related to multiple sclerosis, osteoarthritis, chronic pain, rheumatoid arthritis, gastrointestinal disorders, cardiovascular disorders, and fibromyalgia could also be managed by cryotherapy to avoid contraindications of pharmaceutical treatments for co-morbid insomnia [397, 424, 425]. Cryotherapy could augment antidepressant efficacy in the treatment of depression and present evidence to assess the efficacy of cryotherapy in the synergistic treatment of insomnia [378]. The addition of cryotherapy could enhance recovery during CBT as cryotherapy provided cognitive relief, regulates the HPA axis, and reduce the severity of stress, anxiety, and depression. The synergistic effect of cryotherapy on CBT not only improves cognitive function, memory, and focus but also provides mild relief from depression [426]. Cryotherapy at Hoku point lowered pain during hemodialysis which indicates that a combination of acupressure and cryotherapy has the potential to address the therapeutic need of pain-associated insomnia [427]. TCH efficacy in controlling pain, oxidative stress, inflammation, and circulation of inflammatory molecules could be supported by using cryotherapy resulting in improved outcomes for insomnia treatment. Though the additive /synergistic impact of cryotherapy is promising, more studies are required to establish a comprehensive procedure and conclusion to implicate the efficacy and safety of combinatorial therapy.

**Table 1:** List of pros and cons of various treatment alternatives of insomnia.

Therapy	Pros	Cons	References
**CBT**	Economical, safe, and highly effective	Lack of trained CBT professionals, Need awareness among acceptance of therapy.	[434]
**Pharmacological** **interventions**	Easily accessible, effects are visible in a short time, widely accepted routine therapy	Tolerance, abuse, associated adverse events, and adverse psychological impact	[435, 436]
**TCH**	Effective in the treatment of chronic and incurable diseases.	Lack of clinical trials and pre-clinical studies	[305, 437, 438]
**Acupuncture**	Improves clinical efficacy, Proven traditional therapeutic approach	Lack of consistent data in clinical studies and pre-clinical multiple centers randomized clinical studies	[439, 440]
**PMFT**	Effective in alleviating insomnia, quick reaction time	Safer approach compared to drug therapy	[441]
**Cryotherapy**	Non-invasive, safe, and effective	Adverse events such as erythema, edema, and cold urticaria. availability of expertise, cryo-chamber, and short-term relief	[442, 443]
**Biomaterials and stem cell**	Regenerative therapy, improvement in the delivery of therapeutic agents	Lack of pre-clinical, and clinical studies,	[414, 415, 421, 422]

CBT: Cognitive-Behavioral Therapy, TCH: Traditional Chinese herbs, PMFT: Electro and pulsed magnetic field therapy.

## Limitation of Cryotherapy in Insomnia

Cryotherapy is emerging as a novel non-invasive therapeutic option and studies have reported safe and effective if recommended treatment guidelines are being followed. After cryotherapy, the significant increase in WBC count has been associated with mild side effects such as pain, swelling, redness, blistering, crusting, color alteration, scars, and viral infection in rare cases [428]. Common adverse effects do not require any specific medical attention; however, increased pain at the treatment site, excessive redness, and swelling need medical attention. Cryosurgery could cause transient erythema, edema, and cold urticaria [429], but antihistamines such as cetirizine, loratadine, desloratadine, ebastine, leukotriene receptor antagonists, sulfasalazine, immunosuppressive agents like methotrexate, cyclosporine, omalizumab, and systemic glucocorticoids like prednisolone could be assistive to overcome cold urticaria [430, 431]. Rupatadine, a second-generation antagonist of histamine receptor and platelet-activating factor, at 20mg/day has been found effective and tolerated during acquired cold urticaria [432]. The procedural coherence, availability of expertise, cryo-chamber, and short-term relief are limiting factors for wide acceptability. Though there have been many positive outcomes of cryotherapy as well as its synergistic administration with other alternatives for insomnia, more studies are required to understand the mechanism and establish a uniform therapeutic procedure for the treatment of sleep disorders.

## Key challenges for the treatment of insomnia

Insomnia is one of the most overlooked conditions and is not addressed until it poses serious health risk. There is a wide spectrum of treatment approaches available; however, their selection and effectiveness depend on different factors. The pros and cons of available and prospective therapies are summarized in Table 1. The complex association between insomnia and other comorbidities is not well-understood which poses a challenge in establishing proper treatment [433].

## Conclusions

Recent adverse changes in lifestyle, work culture, and social behavior along with the COVID-19 pandemic situation have exponentially increased stress and other etiological factors which have resulted in disrupted circadian rhythm. The increase in insomnia incidence is moving towards being epidemic which will further deteriorate individual physical and psychological health. Considering the urgency to address this situation, current interventions such as CBT and pharmaceutical therapies supported by lifestyle changes have made considerable progress. In particular, cryotherapy has been reported to reduce oxidative stress, pain, and inflammation resulting in improved sleep efficiency. Novel approaches such as the synergy of cryotherapy and pharmacological/ traditional Chinese medicine/ bioboosti/ electromagnetic and acupressure could provide a more effective way to address the therapeutic needs of insomnia, by suppressing inflammation, oxidative stress, edema, lipid profile, and pain neurotransmission; however, extensive studies are required to establish its broader applicability.
